# Pilot Study on the Use of the “Monocyte-Rich” Platelet-Rich Plasma in Combination with 1927 nm Fractional and 308 nm Excimer Lasers for the Treatment of Vitiligo

**DOI:** 10.3390/medicina57090904

**Published:** 2021-08-30

**Authors:** Santo Raffaele Mercuri, Matteo Riccardo Di Nicola, Pina Brianti, Vittoria Giulia Bianchi, Giovanni Paolino

**Affiliations:** Unit of Dermatology, IRCCS San Raffaele Hospital, 20132 Milan, Italy; mercuri.santoraffaele@hsr.it (S.R.M.); brianti.pina@hsr.it (P.B.); vittoriaabianchi@gmail.com (V.G.B.); paolino.giovanni@hsr.it (G.P.)

**Keywords:** L-PRP, monocytes, xtrac, fraxel, tropocells

## Abstract

*Background and objectives*: Vitiligo is an acquired chronic and idiopathic skin disorder, characterized by selective loss of melanocytes and resulting in a cutaneous depigmentation. Treatment for vitiligo remains a challenge for dermatologists; thus, it is frustrating both for physicians and patients. The objective of this study was to evaluate a combination treatment characterized by the use of a leukocyte-rich platelet-rich plasma, which is particularly rich in monocytes (defined here as monocyte-rich PRP), in combination with a 1927 nm fraxel laser and a 308 nm excimer laser. *Materials and Methods*: Treatment with monocyte-rich PRP combined with 1927 nm fraxel laser and 308 nm excimer laser was performed in nine sessions in 80 days and the median follow-up of the patients was 10 months. A total of 27 Caucasian patients were included in the present study. The median age of patients was 41 years, ranging between 20 and 69 years. *Results*: A re-pigmentation occurred in 16 cases (59%) with a reduction of the Vitiligo Extent Score (VES) and absence of re-pigmentation in untreated areas. Performing a rank correlation between VES and re-pigmentation in the treated areas, we found that there was a significant correlation (*p* < 0.0001). The presence of progressive vitiligo (*p* = 0.1) and the anatomic areas (*p* = 0.1) did not influence the treatment. Untreated areas did not show any improvement of the depigmented lesions, except in one case (*p* < 0.0001). *Conclusions*: in this report, we show for the first time how PRP rich in monocytes, in combination with laser therapies, gives a long therapeutic response, which persists even after 10 months of follow-up.

## 1. Introduction

Vitiligo is definable as an acquired chronic and idiopathic skin disorder, characterized by selective loss of melanocytes and resulting in a depigmentation of skin and hair follicles [1,2,3,4]. Usually, vitiligo lesions result in milky white, often symmetrical macules and patches, with distinct margins; the lesions usually increase in size and number over time and often appear in visible areas like face and limbs. In fair-skinned people, vitiligo is most noticeable in tanned areas, due to the darkening of unaffected skin [1,2]. With an estimated prevalence of 0.5–2% of the population (and local values even much higher, as in some areas of India, where up to 8.8% of the population is affected) [2,3], vitiligo is the most common depigmenting disorder, and it seems to affect both males and females equally, with almost 50% of patients that develop the disease before the age of 20 years and 70–80% before the age of 30 years [3,5,6]. However, the data may be underestimated and the exact values are difficult to detect, also because patients with vitiligo do not always seek medical care [2]. The cause of vitiligo is still not completely understood and different hypotheses regarding its pathogenesis exist [4]; it is a multifactorial disorder characterized by the destruction of functional melanocytes, for which multiple mechanisms have been proposed, such as autoimmune responses, genetic, oxidative stress, the generation of inflammatory mediators and melanocyte detachment mechanisms [3]. Based on these hypotheses, multiple treatments have been developed in order to increase the therapeutic success, reduce relapses and improve patients’ quality of life [4,7]. Corticosteroid, calcineurin inhibitors and 308 nm excimer lasers are involved in active and localized disease treatments, inducing immunosuppression; photo-chemotherapy, ultraviolet B phototherapy and narrowband ultraviolet B phototherapy are also valid treatments for generalized vitiligo [4,8]. Instead, surgery techniques are mainly based on autologous transplanting of melanocytes [4,9,10]. Despite the presence of several therapeutic options, they all have shown limited responses [4,7] and vitiligo treatment is still considered one of the most difficult dermatological challenges [3]. However, this should not justify a lack of positivity in the clinical management of the problem: patients who seek a cure for vitiligo are often told that there are no solutions and that they must learn to live with that disorder, which is a discouraging attitude for patients [2]. They should instead feel motivated by the proposal of existing treatments that at least manage to maintain or even improve the status of vitiligo, which is not just a cosmetic disease, but also can give patients heavy psychological repercussions [3,11].

### 1.1. 308 nm Excimer and 1927 nm Fractional Laser Treatments

A 308 nm excimer laser is an ultraviolet B radiation system with a monochromatic light at a specific wavelength and it is formed by dimerides of noble gas and halide after activation and a continuous pulsatile release under the action of an electric current [12,13,14]. Its use in the treatment of vitiligo is well documented, both as a single treatment and in combination with other procedures [14,15,16,17,18,19,20,21,22,23,24,25,26,27,28,29,30,31,32,33]. The exclusive use of a 308 nm excimer laser involves very long treatment times and long-term irradiation poses a potential risk of carcinogenesis, while a combined treatment improves the effect on vitiligo, shortens the treatment course, and reduces laser accumulation [12,14].

1927 nm fractional laser is a fractional thermolysis device which features a thulium fiber laser with a 1927 nm wavelength, with ablative and non-ablative capabilities. It allows to treat both the epidermal and dermal layers [34,35]. Its particular wavelength has a high-water absorption coefficient, allowing it to intervene on epidermal processes such as pigmentation and discoloration. This laser is particularly suitable for surface treatments of the epidermis, reaching a penetration of 200 μm, compared to 1400–1500 μm for a wavelength of 1550 nm of common erbium lasers [35]. Different wavelength fractional lasers are documented in the treatment of vitiligo, both as a single treatment and in combination with other procedures [36,37,38,39,40,41]. The 1927 nm fractional laser is known for other several dermatological treatments such as cosmetic rejuvenation [42], contrast of photoaging [35,43], treatment of actinic cheilitis [44], seborrheic keratosis [45], melasma [35], disseminated superficial actinic porokeratosis [46] and contracture of scars [47]. Specifically, the fractional 1927 nm laser, by targeting water as its chromophore, induces a dense array of microscopic, columnar thermal zones of tissue injury, extending down to a depth of 300 μm. This leads to gentle photothermal damage. Accordingly, in order to allow for a greater and more direct penetration of PRP in the skin through the gentle cutaneous pores, the fractional 1927 nm laser was added in the current protocol.

### 1.2. Platelet-Rich Plasma (PRP)

Platelet-rich plasma (PRP) is an autologous preparation of concentrated platelets of plasma that contains different growth factors that, in the context of vitiligo, are hypothesized to promote stimulation of melanocytes [3]. PRP increases the release of growth factors, adhesion molecules and chemokines as well as promotes cell differentiation, proliferation and regeneration [4,48]. For an overview of the main growth factors presented in PRP, see the reviews of Hesseler and Shyam, Alves and Grimalt and Mercuri et al. [4,48,49]. Preliminary studies have shown an ineffectiveness of PRP alone [3,50], but subsequent works have highlighted the effectiveness of PRP used in combination with other treatments. For example, Ibrahim et al. used PRP on the outcome of short-term NB-UVB therapy for patients with stable vitiligo, demonstrating better results than using the NB-UVB alone [3,4,51]; Abdelghani et al. have shown benefits in the combined use of a fractional CO_2_ laser and PRP [4,38]; Kadry et al. have shown benefits in the use of both PRP alone and PRP combined with a fractional CO_2_ laser [4,40]; Khattab et al. have shown positive results with the use of PRP and an excimer laser [3,33]. There are different types of medical devices to obtain PRP, produced by various pharmaceutical companies. Basically, the devices vary according to the type of preparation (centrifugation speed and use of anticoagulant), the percentage of platelets, leukocytes and growth factors and according to the applications to which they are addressed [49,52]. Different authors have tried to categorize the various types of PRP, but, in fact, there is still no uniquely recognized classification that allows us to accurately compare the effectiveness of different devices and different studies [49]. An often-used classification is the one of Dohan Ehrenfest et al. [4,49,53], which proposes a division into four categories based on the presence/absence of a cell content and the fibrin architecture:

(a) Pure platelet-rich plasma: a PRP poor in leukocytes, with a low-density fibrin network after activation;
(b) Leukocyte-rich platelet-rich plasma: a PRP rich in leukocytes, with a low-density fibrin network after activation;
(c) Pure platelet-rich fibrin: a PRP poor in leukocytes, with a high-density fibrin network (unlike the previous ones, this cannot be injected and exists in an activated gel form);
(d) Leukocyte- and platelet-rich fibrin: a PRP rich in leukocytes, with a high-density fibrin network.

This classification has some critical issues, such as the lack of data on the final volume of the preparation, the presence or absence of red blood cells in the PRP and the dose of platelets in the PRP obtained [49]. A more complete classification is the DEPA (*Dose* of injected platelets; *Efficiency* of the production; *Purity* of the PRP obtained; *Activation* process), proposed by Magalon et al., which is based on the quantity of platelets obtained by the PRP devices, on product purity and on platelet activation before the injection. The parameters considered by the DEPA are exhaustive but cannot be measured by clinicians, so they should be declared in the characteristics of each medical device [49,54,55,56,57,58].

### 1.3. Monocyte-Rich Platelet-Rich Plasma

This study reports the first evidence on the use of a leukocyte-rich platelet-rich plasma with a relatively high concentration of monocytes (hereinafter defined as monocyte-rich PRP), in combination with both 1927 nm fractional and 308 nm excimer lasers, for the treatment of vitiligo. The preparation system used to obtain the monocyte-rich PRP is the Tropocells^®^ PRP (Estar Medical, Holon, Israel). According to Kobayashi et al. [55], a standard leukocyte-rich platelet-rich plasma contains the following: 846.5 ± 431.8 × 10^3^/µL platelets; 3.2 ± 1.2 × 10^3^/µL erythrocytes; 14.9 ± 4.5 × 10^3^/µL leukocytes (5.4 ± 3.0 × 10^3^/µL neutrophils; 8.1 ± 1.9 × 10^3^/µL lymphocytes; 1.4 ± 0.6 × 10^3^/µL monocytes). Otherwise, the monocyte-rich platelet-rich plasma contains the following: 947 × 10^3^/µL platelets; 0.03 × 10^3^/µL erythrocytes; 3.67 × 10^3^/µL leukocytes (0.6 × 10^3^/µL neutrophils; 1.73 × 10^3^/µL lymphocytes; 1.34 × 10^3^/µL monocytes).

Then, according to the data provided by the manufacturer, the monocyte-rich PRP kit is able to obtain a platelet-rich plasma with a high concentration of peripheral blood mononuclear cells (with a proportionately lower amount of lymphocytes), a low concentration of neutrophils and an almost complete depletion of red blood cells. We decided to test monocyte-rich PRP in vitiligo, since peripheral monocytes demonstrated regenerative effects in the skin through the activation of macrophage colony-stimulating factors and because at the same time, as L-PRP, it may influence epidermal pigmentation, through a direct activity on melanin distribution/degradation and through activation of keratinocytes for the production of melanogenic factors [58].

## 2. Materials and Methods

**Figure 1:**
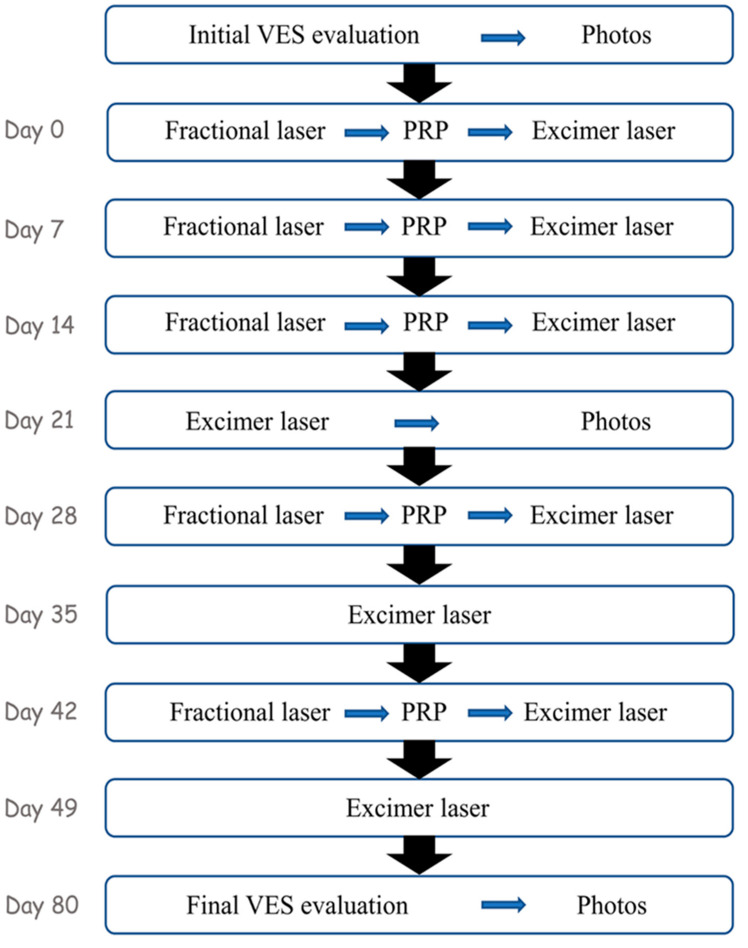
Flowchart of treatments and activities carried out in the 10 sessions.

**Figure 2:**
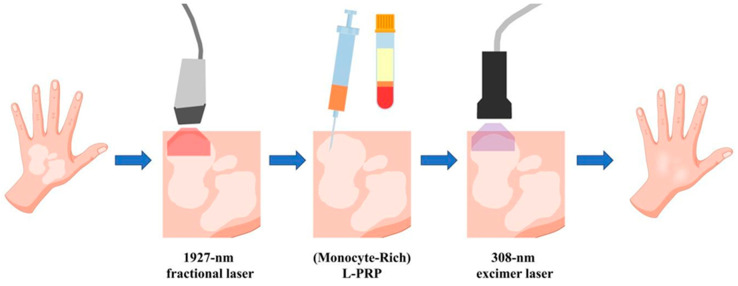
Scheme of the steps carried out in a complete vitiligo treatment session. L-PRP means leukocyte-rich PRP. Vector files downloaded from https://www.vecteezy.com.

To perform this study we selected patients with an age ≥18 years; for each patient, a single patch of vitiligo with a maximum diameter of 10 cm was selected for treatment. Other cutaneous lesions were not treated and used as controls. Candidates must not have had any other excimer/fractional laser treatments in the past and they must have been off any topical therapy for at least 6 months; they were not to have thrombocytopenia (with a blood platelet value of less than 150,000 plt/μm) or to be suffering from celiac disease, thyroiditis or autoimmune systemic diseases; they were not to be pregnant or lactating women. The study was approved by the local Ethic Committee and registered with the acronym “PG-FRAXEL”, and all patients signed informed consent for taking part in the study. The therapeutic program included 10 sessions for each patient (Figure 1) starting with an evaluation of the disease through the Vitiligo Extent Score (VES) [56] and an initial photographic documentation. In the second session, the first series of treatments was carried out (Figure 2): a treatment with a 1927 nm fractional laser (Fraxel re:store DUAL, Solta Medical, Hayward, CA, USA) was performed in involved areas with 20 mJ/mb at 4 W, 8 MTZ/cm^2^, resulting in 2.5–3.0% coverage and 500 mJ/cm^2^ total energy delivered per pass. The patient was treated with 8 passes. Subsequently, an application/injection of the PRP preparation (obtained through the Tropocells^®^ PRP kit (Estar Medical, Holon, Israel)) was performed and, after 10 min, a treatment with the 308 nm excimer laser (XTRAC^®^ PhotoMedex, Horsham, PA, USA) was performed at an intensity of 100 mJ/cm^2^.

In the third and fourth sessions, the same procedures as for the second session were repeated 7 days apart. In the fifth session (21 days from the first treatment), only the therapy with the excimer laser and a photographic documentation of half the treatment was carried out.

The sixth session (28 days after the first) included a further complete treatment with the fractional laser + PRP and the excimer laser. In the seventh session (35 days after the first), only the excimer laser was applied. The eighth session (42 days after the first) included the complete treatment, while the ninth session (49 days after the first treatment) only included the excimer laser. The tenth and final session (31 days from the last, therefore 80 from the first) included the final VES evaluation and the final photographic documentation.

The response or lack of response to the treatment was arbitrarily performed on a dichotomous assessment scale by an independent physician, characterized by a range between ≥60% or ≤59%. In order to have a more objective clinical evaluation, the score was calculated after a follow-up of 10 months. For the statistical analysis, a rank correlation was performed in order to evaluate any correlation between single variables and possible response to treatments. A *p*-value < 0.05 was considered statistically significant.

## 3. Results

A total of 27 Caucasian patients were included in the present study. The median age of patients was 41 years, ranging between 20 and 69 years. Regarding the gender, 7 patients were male and 20 were females, and regarding the comorbidities, 2 patients had thyroiditis, 3 patients hypertension and 1 patient a type II diabetes. Six patients had a positive familiar history for vitiligo. All patients involved in the study showed a stable and nonsegmental vitiligo.

**Figure 3:**
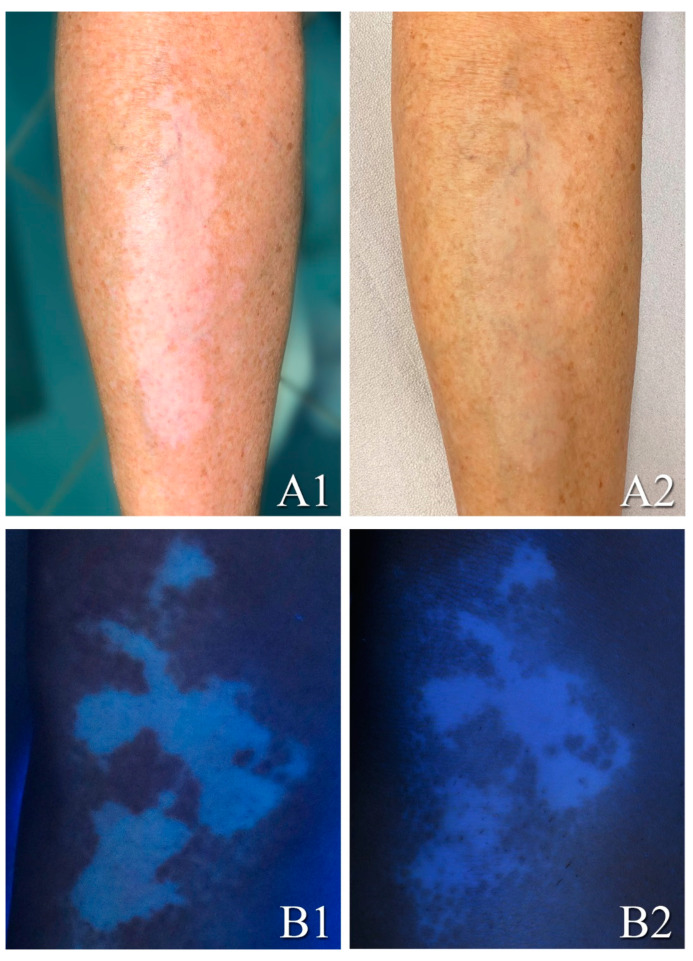
Examples of vitiligo lesion before and after full treatment in two different patients after a follow-up of 10 months, under a standard light lamp (**A1**,**A2**) and under a UV lamp (**B1**,**B2**): vitiligo lesion in the tibia area (**A1**) improved following moderate uniform re-pigmentation (**A2**); vitiligo lesion in the knee area (**B1**) improved following a point re-pigmentation (**B2**).

The median VES at T0 was 0.65 (ranging between 0.13 and 12), while the median VES after 10 months of follow-up T1 was 0.4 (ranging between 0 and 11). The mean VES at T0 was 2.40, while the mean VES after 10 months of follow-up T1 was 2.21. The study aimed to evaluate treatment in a single patch area, by assessing re-pigmentation employing a dichotomous scale to a single treated area (according to the presence or absence of a re-pigmentation ≥60 for a single area); we found that a re-pigmentation occurred in 16 cases (59% of total) (Figure 3). The patients with re-pigmentation were the same that showed a reduction of VES score. The general VES variation, however, which relates to other untreated body areas and reflects the overall vitiligo involvement of the body (extent), was obviously not significant. Regarding side effects, all patients experienced only a mild local burning sensation and erythema that disappeared in 3 days. Performing a rank correlation between the VES and the dichotomous scale evaluation of treatment, we found that there was a significant correlation (*p* < 0.0001; 95% IC = 0.714 to 0.935) with a Spearman’s coefficient of rank correlation (rho) equal to 0.860.

In total, 7 (*n* = 7) out of 16 patients that showed a response to the treatment had a progressive vitiligo (43%; *p* = 0.1), demonstrating how the progression of the disease does not affect therapy. At the same time, 7 out of 16 patients that showed a response to the treatment had an involvement of the limbs (43%; *p* = 0.1), of which 6 had an involvement of the arms/legs and 1 had a hands involvement. Contrariwise, the other anatomic areas affected by vitiligo that were not treated did not show any improvement of the depigmented lesions, except in one case (*p* < 0.0001; 95% IC = 0.623 to 0.915). Clinical features are summarized in Table 1.

## 4. Discussion

The cause of vitiligo is not yet fully understood. Several hypotheses regarding the pathogenesis of the disease exist, although none has been confirmed as the main one. These main hypotheses include the autoimmune hypothesis, neural hypothesis, self-destruct hypothesis and biochemical hypothesis [4]. Based on each of these hypotheses, various treatments have been developed in the last decades, in order to increase therapeutic success in reducing relapses and improving patients’ life quality. Corticosteroid, calcineurin inhibitors and 308 nm excimer lasers play a role in active and localized disease, inducing immunosuppression. At the same time, photo-chemotherapy, UVB phototherapy and narrowband UVB phototherapy are also other valid treatments for generalized vitiligo [4], while surgery techniques are mainly based on autologous transplanting of melanocytes. However, despite the presence of several therapeutic options, all these therapeutic modalities show limited responses [4]. Recently, Khattab et al. [33] included in a study 52 patients (8M:44F) with stable, nonsegmental and symmetrical vitiligo, randomized in two different groups, in order to evaluate the efficacy of the treatment with combined excimer laser and PRP. Specifically, in the first group, the patients were treated with an intradermal PRP injection and excimer laser (PRP after excimer laser every 3 weeks for a total of six sessions of PRP), while in the second group, the patients were treated with only an excimer laser. A better response was reached in the first group, also according to the VES score. Specifically, in Khattab et al.’s study [33], group I showed an excellent re-pigmentation in 34.6% of patients and a good re-pigmentation in 50% of patients, while in group II, no patients showed an excellent response, with only 34.6% of patients that showed a good response. In this context, our results are closer to those obtained by group I of the study of Khattab et al. [33], showing a therapeutic response (≥60% of vitiligo areas) in 59% of cases.

To our knowledge, the “monocyte-rich” PRP was used for the first time for the treatment of vitiligo. In this study, evaluating the individual areas treated and voluntarily dividing the patients into a dichotomous scale (according to the presence or absence of a re-pigmentation ≥60% for a single treated area), we found that a re-pigmentation of the vitiligo area was present in 16 cases, or 59% of cases. The strength of the response in our sample lies in the three methods used and their different mechanisms of action. Specifically, at first, a 1927 nm fractional laser (Fraxel re:store DUAL, Solta Medical, Hayward, CA, USA) treatment was performed in involved areas with 20 mJ/mb at 4 W, 8 MTZ/cm^2^, resulting in 2.5–3.0% coverage and 500 mJ/cm^2^ total energy delivered per pass. The patients were treated with eight passes. In this regard, the fractional laser allowed for the formation of gentle cutaneous pores, which favor the penetration of L-PRP, leading to a greater penetration on the skin of PRP. L-PRP has a 6-fold platelet concentration and increases PDGF-AB and TGF-β1 concentrations compared with whole blood, and also strongly activates the NF-κB pathway and contains a high amount of leukocytes, TNF-α and IL-1β [57]. The secretion of these factors correlates with the hypothesis of some authors, who consider that L-PRP contributes to inflammation and premature apoptosis [58]. Furthermore, one study argued that L-PRP may intensify the over-expression of MMP and consequently activate extracellular matrix (ECM) catabolic pathways and excessive inflammation [59]. This concept was also accentuated in another study [60], which reported that leukocytes in PRP initiate a greater activation of the nuclear factor kappa-light-chain-enhancer of the activated B cells pathway, subsequently resulting in significantly less fibroblast proliferation and a higher concentration of pro-inflammatory cytokines [58,59,60]. However, the PRP of these studies contained a high concentration of RBCs and therefore could be the source of this reaction and bias [58]. At the same time, L-PRP enhances fibroblast cell proliferation and cell migration and demonstrated either an up-regulation or down-regulation gene expression profile of the extracellular matrix and adhesion molecules [58]. These molecules, in conjunction with growth factors and leukocytes, generate an ideally organized ECM infrastructure, which synchronizes cell growth [58]. Fibroblasts, the extracellular matrix and adhesion molecules influence epidermal pigmentation, either through a direct activity on melanin distribution/degradation or activation of keratinocytes for the production of melanogenic factors [61]. It follows that, as reported in our pivotal study, their activation may induce a greater therapeutic response in re-pigmentation in patches of vitiligo. In our L-PRP, there was a high content of monocytes; peripheral blood monocytes propagate pluripotent stem cells, which are subsequently induced by the macrophage colony-stimulating factor and also transform into anti-fibrotic keratinocyte-like cells, and therefore demonstrate regenerative effects [58]. Finally, the 308 nm excimer laser (XTRAC^®^ PhotoMedex, Horsham, PA, USA) treatment at an intensity of 100 mJ/cm2 was performed. Targeted phototherapy with the 308 nm excimer laser delivers UV radiation to the affected area only [17]. This limits total skin exposure to UV radiation and therefore may decrease the risk of skin cancer. Besides, the 308 nm excimer laser presents many advantages compared with NB–UV-B therapy for treating localized vitiligo [22]. Photobiological effects seem more effective because the induction of lymphocyte apoptosis is superior [22]. The combined treatment of fractional laser, PRP and excimer laser has undoubtedly improved the clinical response in the treated patients. A limitation of the study is casuistry; unfortunately, having started in the period of the COVID-19 pandemic, the study suffered from a reduction in the number of patient adhesions, as well as carrying out a case–control study in general.

## 5. Conclusions

Treatment for vitiligo remains a challenge for dermatologists. The combination of one or more therapeutic methods may improve the therapeutic response in patients; for this reason, every new therapeutic technique must be evaluated and presented to the scientific community to broaden the therapeutic spectrum in this class of patients. In this report, we show for the first time how the so-called monocyte-rich PRP, together with laser therapies, may give a long therapeutic response, which persists even after 10 months of follow-up.

## Figures and Tables

**Table 1:** Clinical features.

	*n*	*p* *
Gender Male Female	7 20	0.1
Age	41	-
Anatomic area Hands/Feet Arms/Legs Trunk/Head/Neck	5 7 15	0.1
Comorbidities Yes No	6 21	0.3
VES T_0_ VES T_1_	0.65 0.4	<0.0001
Re-pigmentation TA Yes No	16 11	-
Re-pigmentation NTA Yes No	1 26	<0.0001

VES = Vitiligo Extent Score; re-pigmentation = dichotomous assessment scale by an independent physician, characterized by a range between ≥ 60% and ≤ 59%: p * = rank correlation was performed in order to evaluate any correlation between single variables and possible response to treatment; re-pigmentation TA = re-pigmentation in the treated areas; re-pigmentation NTA = re-pigmentation in untreated areas.
